# Supplementary material for: Effectiveness and Cost-Effectiveness of Self-Management Interventions for Adults Living with Heart Failure to Improve Patient-Important Outcomes: An Evidence Map of Randomized Controlled Trials
Source: Healthcare (Basel). 2024 Jan 24;12(3):302. doi: 10.3390/healthcare12030302 (PMC10855227; doi:10.3390/healthcare12030302)
Supplement: Supplementary file 1 [file healthcare-12-00302-s001.zip › healthcare-2733421-supplementary.pdf]

Supplemantary S1

Search strategies

Database	Numbers
Pubmed	2240
Embase	1766
Cinahl	2369
PsycINFO	113
Cochrane	2068
Total	8556
After duplication	**6057**

Draft

Search History Pubmed 8 december 2018

Search	Query	Items found
#3	#1 AND #2 AND #3 Filters: Publication date from 2010/01/01	2240
#4	#1 AND #2 AND #3	4240
#3	("Clinical Trials as Topic"[Mesh] OR "Randomized Controlled Trial" [pt] OR "Controlled Clinical Trial" [pt] OR randomized [tiab] OR randomly[tiab] OR placebo[tiab] OR trial[ti]) NOT (animals[mh] NOT humans[mh])	1167356
#2	"Self Care"[Mesh] OR "Self-Management"[Mesh] OR "Power (Psychology)"[Mesh] OR "Health Education"[Mesh] OR "Patient Participation"[Mesh] OR "Decision Making"[Mesh] OR “Telemedicine”[Mesh] OR self-administration*[tiab] OR self-care[tiab] OR self efficac*[tiab] OR self-manag*[tiab] OR selfmanag*[tiab] OR self-monitor*[tiab] OR selfmonitor*[tiab] OR self-diagnos*[tiab] OR selfdiagnos*[tiab] OR self-assess*[tiab] OR selfassess*[tiab] OR self-direct*[tiab] OR selfdirect*[tiab] OR self-help*[tiab] OR empower*[tiab] OR enablement[tiab] OR health education[tiab] OR patient education[tiab] OR patient participation[tiab] OR coach*[tiab] OR health promot*[tiab] OR ((community[tiab] OR peer[tiab]) AND (support*[tiab] OR advice*[tiab] OR monitor*[tiab] OR train*[tiab] OR instruction*[tiab] OR intervention*[tiab] OR consult*[tiab] OR assist*[tiab] OR educat*[tiab] OR information*[tiab] OR skill*[tiab])) OR group support*[tiab] OR group intervention*[tiab] OR group advice*[tiab] OR group monitor*[tiab] OR group train*[tiab] OR	1904809

Search	Query	Items found
	training group*[tiab] OR group instruct*[tiab] OR group assist*[tiab] OR group educat*[tiab] OR group inform*[tiab] OR group skill*[tiab] OR ((patient[tiab] OR patients[tiab]) AND (centered[tiab] OR centred[tiab] OR focus*[tiab] of directed[tiab] OR coach*[tiab] OR engage*[tiab] OR involve*[tiab] OR orient*[tiab] OR participat*[tiab])) OR ((patient[tiab] OR patients[tiab]) AND (educat*[tiab] OR train*[tiab] OR instruct*[tiab] OR teach*[tiab])) OR (management*[tiab] AND (plan[tiab] OR program*[tiab] OR disease[tiab])) OR (symptom*[tiab] AND (management[tiab] OR directed[tiab] OR focus*[tiab])) OR ((personalized[tiab] OR personalized[tiab]) AND care[tiab]) OR telemedicine[tiab] OR eHealth[tiab] OR e-Health[tiab] OR mHealth[tiab] OR m-Health[tiab] OR shared decision*[tiab] OR sharing decision*[tiab] OR informed decision*[tiab] OR informed choice*[tiab] or decision aid*[tiab] OR ((share*[ti] OR sharing*[ti] OR informed*[ti]) AND (decision*[ti] OR deciding*[ti] OR choice*[ti]))	
#1	"Heart Failure"[Mesh] OR heart failure[tiab] OR cardiac failure[tiab] OR myocardial failure[tiab]	193558

Supplementary S2

Eligibility criteria

Draft

Inclusion criteria	Considerations
1. The article is in an eligible format and language	The article should be written in English or Spanish.<br><br>Invalid article types such as study proposals or designs as well as dissertations should be excluded.
2. Type of study is a randomized controlled trial.	If at the full-text assessment or data extraction phase you encounter a study labelled as quasi-random, quasi-randomized, or quasi-experimental, please:<br>1. Before excluding, check whether the study actually meets the methodological criteria for being classified as an RCT (i.e. it is a prospective study, it compares two or more interventions (one or more can be control/no treatment), and there is an allocation sequence that is generated via a random or quasi-random system). Some studies may self-classify as quasi-experimental/random based on not implementing a sample size calculation or for evening out baseline characteristics, e.g. gender or age; none of these are grounds for excluding based on study design.<br>2. Studies that recruit a convenience sample can be included.

Draft

3. Participants are all 18 years or older, or at least 80% is 18 years or older.	
The target population is people diagnosed with one of the following:<br>● Heart failure (and specifications of this, like Right-Sided Heart Failure, Left-Sided Heart Failure, Congestive Heart Failure, Diastolic Heart Failure, Systolic Heart Failure)<br>● Cardiac Failure<br>● Decompensation of Heart. Decompensation Cordis<br>● Myocardial Failure<br>● Chronic heart failure	At least 80% of the sample should have one of these diagnoses.<br><br>**Exclude:** myocardial infarction and ischemic heart disease, as they may be a cause of heart failure but are not heart failure itself<br><br>**Pregnant women are excluded**
4. Type of population is patients or caregivers.	If the study population are professionals or providers exclude study. Note: if the study targets professionals and patients simultaneously, also exclude study.<br>**Caution**: it might be that in the INTERVENTION description they mention that professionals were also trained to carry out the intervention. This is NOT a reason for exclusion.
5. It's a self-management intervention<br><br>**Self-management** is defined as "what individuals, families and communities do with the intention to promote, maintain, or restore health and to cope with illness and disability with or without the support of health professionals. It includes but is not limited to **self-prevention, self-diagnosis, self-**	An SMI should have as its core goal to increase patient autonomy, by INCREASING patient's knowledge, skills and/or changing its behaviors (outside direct clinical supervision).<br>An intervention is usually carried out by a professional (sometimes by peers or other lay persons).

**medication and self-management of illness and disability**...".).<br><br>› **<u>Self-management interventions</u>**<br>SMI's are systematically **provided by healthcare staff or other patients or lay persons** to increase patients' skills and confidence in managing their chronic condition.<br>SMI's **aim to equip patients** (and their informal caregivers whenever appropriate) to actively participate in the management of their chronic condition.	However, if an intervention is always carried out under direct supervision of a clinician it should be excluded.<br><br><u>Common areas of doubts:</u><br>**Physical activity**<br>- If a patient carries out a physical activity always under clinical supervision, this is not considered and SMI<br>- whereas if they train patients to carry out a set of physical activities in their daily live, this would be considered an SMI<br>- If an intervention involves both supervised and unsupervised exercise, we include if roughly **at least half** of the exercise sessions are unsupervised. If patients practice at home together with a family member this is still unsupervised. Supervised is under professional supervision. Practicing at home under supervision of a nurse or physiotherapist therefore still counts as supervised<br><br>**Telemonitoring:**<br>- If data is transmitted automatically, without any actions needed from the patient, this is not self-management<br>- If patients do need to self-monitor and record their clinical values themselves, this counts as self-management<br>**Diet**

Draft

Draft

	- If a study measures the effectiveness of a diet under laboratory conditions, this is NOT considered an SMI<br>- If a study trains patients to carry out a healthy diet in their daily life, this would be considered an SMI.
6. The self-management intervention should be the **<u>main focus</u>** of the intervention.	If the self-management intervention is not the more central component, it's just one of many other components ⊚ exclude study. For example, if an intervention is introducing a disease management program, which includes elements of SMI but has stronger component of organizational change, it should be EXCLUDED.<br><br>If the self-management intervention is compared with other components but outcomes are reported separately, it should be included.<br>For example, if in a case of disease management program the authors independently analyze the effectiveness of the SM components, the study can be included.
7. The RCT includes, at least, one of our outcomes of interest for heart failure:<br>● Knowledge<br>● Health literacy<br>● Self-efficacy	

● Patient activation<br>● Self-monitoring<br>● Adherence to medication or other treatment<br>● Adherence to diet as agreed (including salt and water)<br>● Weight management<br>● Breathlessness (Dyspnea)<br>● Exercise capacity (including effort test)<br>● Physical activity<br>● Swelling (including leg and abdominal edema)<br>● Mortality<br>● Quality of life<br>● Caregiver quality of life<br>● Perception of health care professional relationship and communication<br>● Participation and decision making<br>● Healthcare use (including the number of (re-)hospitalizations and emergency hospital visits)<br>● Healthcare costs	
8. Not an inclusion criterion, but if a study is relevant for another of our target diseases, flag it in Covidence. You can use the following criteria:<br><br>**Diabetes:** participants have diagnosed diabetes Type 2, or if it's a mixed population of Type 1 and Type 2 patients at least 80% has Type 2.<br><br>**Obesity:** inclusion criteria states that participants have a BMI of 25 or higher (or 23 for Asian populations) **OR** at least 97.5% has a BMI of 25 or higher, **OR** mean – 2*SD amounts to 25 or higher.<br><br>**COPD:** patients should be diagnosed with one of the following:<br>● COPD or COAD<br>● Chronic Obstructive Pulmonary Disease<br>● Chronic Obstructive Airway Disease<br>● Chronic Obstructive Lung Disease<br>● Chronic Airflow Obstruction<br>● (Chronic) Bronchitis<br>● Pulmonary Emphysema<br>● Emphysema	

Draft

Draft

Supplementary S3

Heart failure: description of outcomes and tools

Outcome	N studies	Name tool	N tools
Adherence to medication	37		
Adherence to medication		Researchers self-developed questionnaire	9
Adherence to medication		Morisky Medication Adherence Scale-8 (MMAS-8),	5
Adherence to medication		Medication Event Monitoring System (MEMS)	4
Adherence to medication		MOSSAS-3HF	3
Adherence to medication		Medication Adherence Report Scale (MARS)	2
Adherence to medication		Brief Medication Questionnaire (BMQ)	1
Adherence to medication		Hill-Bone compliance scale	1
Adherence to medication		Dutch Heart Failure Knowledge Scale	1
Body weight	13		
Weight gain		Item Checklist of CHF Symptoms	1
Dyspnea	15		
Dyspnea		Dyspnea/Fatigue Index	3
Dyspnea		Item Checklist of CHF Symptoms	2
Dyspnea		Congestive Heart Failure Questionnaire (CHFQ) - dyspnea subscale	2
Dyspnea		Fatigue Severity Scale (FSS)	1
Dyspnea		Edmonton Symptom Assessment System (ESAS)	1
Dyspnea		Medical Research Council scale (MRC)	1
Dyspnea		Shortness of Breath Visual Numeric (SBVN)	1
Dyspnea		Researchers self-developed questionnaire	1
Caregiver quality of life	6		
Caregiver quality of life		Caregiver Burden Questionnaire for HF (CBQ-HF)	2
Caregiver quality of life		HADS - depression subscale	2
Caregiver quality of life		HADS - anxiety subscale	2
Caregiver quality of life		Family Caregiver Quality of Life Scale Questionnaire (FAMQOL)	1
Caregiver quality of life		Zarit burden interview (ZBI)	1
Caregiver quality of life		Impact of Informal Caring Scale, Care WorkStrain Subscale	1
Caregiver quality of life		Center for Epidemiologic Studies Depression Scale (CES-D)	1
Caregiver quality of life		Caregiver Burden Scale - total score	1
Caregiver quality of life		Memorial Symptom Assessment Scale-HF (MSAS-HF)	1

Draft

Caregiver quality of life		Profile of Mood States (POMS)	1
Caregiver quality of life		Caregiver Quality of Life Index	1
Exercise capacity (including effort test)	71		
Exercise capacity (including effort test)		6-min walking test (6MWT)	46
Exercise capacity (including effort test)		New York Heart Association (NYHA) Class of chronic HF	15
Exercise capacity (including effort test)		Incremental Shuttle Walking Test (ISWT)	5
Exercise capacity (including effort test)		Cardiopulmonary Exercise Test (CPET)	2
Exercise capacity (including effort test)		Short Physical Activity Battery (SPPB)	2
Exercise capacity (including effort test)		Duke Activity Status Index (DASI)	2
Exercise capacity (including effort test)		Accelerometer	2
Exercise capacity (including effort test)		Borg Rating of Perceived Exertion scale	2
Exercise capacity (including effort test)		10-Meter Walking Test (10MWT)	1
Exercise capacity (including effort test)		Balance Outcome Measure for Elder Rehabilitation (BOOMER)	1
Exercise capacity (including effort test)		Continuous Scale Physical Functional Performance Test (CS-PFP10 or CS-PFP16)	1
Exercise capacity (including effort test)		Researchers Self-developed Questionnaire	1
Exercise capacity (including effort test)		2-min walking test (2MWT)	1
Exercise capacity (including effort test)		Specific Activity Scale	1
Knowledge	37		
Knowledge		Researchers Self-developed Questionnaire	19
Knowledge		Dutch Heart Failure Knowledge Scale (DHFKS)	13
Knowledge		Atlanta Heart Failure Knowledge Test (AHFKT)	2
Knowledge		Atlanta Heart Failure Knowledge Test - Version 2 (AHFKT-v2)	2
Knowledge		Knowledge Acquisition Questionnaire (KAQ)	1
Participation and decision making	1		
Shared decision-making		The Degner Control of Preferences Scale	1
Shared decision-making		Satisfaction with Decision Scale (SWD)	1
Shared decision-making		Decision Regret Scale (DRS)	1
Shared decision-making		CollaboRATE	1
Patient activation	3		
Patient activation		Patient Activation Measure (PAM)	2
Patient activation		Power as Knowing Participa-tion in Change Tool VII	1
Perception of health care professional relationship and communication	4		
Perception of health care professional relationship and communication		Communication with Physicians (CP)	1

Perception of health care professional relationship and communication		Quality of communication (QOC)	1
Perception of health care professional relationship and communication		Hospital Consumer Assessment of Healthcare Providers and Systems. (HCAHPS)	1
Physical activity	14		
Physical activity		Physical Activity Scale for the Elderly (PASE)	4
Physical activity		CHAMPS (Community Healthy Activities Model Program for Seniors) physical activity questionnaire	3
Physical activity		Researchers self-developed questionnaire	2
Physical activity		Physical Activities Scale - walking subscale	2
Physical activity		Groningen Activity Restriction Scale	1
Quality of life	161		
Quality of life		Minnesota Living with Heart Failure Questionnaire (MLHFQ)	87
Quality of life		Kansas City Cardiomyopathy Questionnaire (KCCQ)	24
Quality of life		Short Form 36 Health Survey (SF-36)	18
Quality of life		EuroQoL (EQ-5D-5L) - generic HRQoL	10
Quality of life		EuroQol Visual Analogue Scale (EQ-5D VA)	8
Quality of life		Researchers Self-developed Questionnaire	6
Quality of life		Heart Related Quality of Life (HeartQoL)	3
Quality of life		Chronic Heart Failure Questionnaire (CHQ)	2
Quality of life		Left Ventricular Dysfunction Questionnaire (LVD-36)	1
Quality of life		Life Satisfaction Questionnaire Z-version (LSI-Z)	1
Quality of life		Iranian Heart Failure Quality of Life Questionnaire (IHF-QOL)	1
Quality of life		MacNew Heart Disease Health-related Quality of Life Instrument	1
Quality of life		Hospice QOL Index (HQLI)	1
Quality of life		The Memorial Symptom Assessment Scale-HF	1
Quality of life		Functional Assessment of Chronic Illness Therapy Spiritual Well-Being (FACIT-Sp)	1
Quality of life		Care Dependency Scale (CDS)	1
Quality of life		Heart Failure Symptom Scale (HFSS)	1
Quality of life		Kansas City Cardiomyopathy Questionnaire (KCCQ)	1
Quality of life		SF-8 Health Survey	1

Draft

Draft

Quality of life		McGill Quality of Life Questionnaire (MQOL)	1
Quality of life		Perception of General Well-Being Inventory (PGWBI)	1
Quality of life		Nottingham Health Profile (NHP)	1
Quality of life		Quality of Well-Being - Self-administered (QWB-SA)	1
Quality of life		Quality of Life Index (QLI)	1
Quality of life		EuroQol Visual Analogue Scale (EQ-5D VA)	1
Self-efficacy	68		
Self-efficacy		Self-care Chronic Heart Failure Index (SCHFI)	34
Self-efficacy		European Heart Failure Self-care Behavior Scale (EHFScBS-9)	17
Self-efficacy		Researchers Self-developed Questionnaire	6
Self-efficacy		Barnason Efficacy Expectation Scale(BEES-HF) 16 item or 15 item version	2
Self-efficacy		Therapeutic Self-Care Scale	2
Self-efficacy		Self-efficacy for managing chronic diseases scale (SEMCD)	2
Self-efficacy		General Self-efficacy Scale (GSES)	2
Self-efficacy		Bandura exercise self-efficacy scale	2
Self-efficacy		Heart Failure Self-Care Behaviour Scale (EHFScBS-9)	2
Self-efficacy		Perceived Competence Scale (PCS)	2
Self-efficacy		Self-Management of Heart Failure (SMHF) scale	1
Self-efficacy		Cardiac Exercise Self-efficacy Instrument	1
Self-efficacy		Caregiver Demands Scale (CDS)	1
Self-efficacy		Cardiac Self-Efficacy Scale	1
Self-efficacy		Heart Failure Self-Efficacy Scale (HFSE-30)	1
Self-efficacy		Confidence and Conviction scale	1
Self-efficacy		Appraisal of Self-care Agency (ASA) Scale	1
Self-monitoring	6		
Self-monitoring		Researchers Self-developed Questionnaire	1
Self-monitoring		Health Education Impact Questionnaire (heiQ) - subscale	1
Swelling	2		
Swelling		Item Checklist of CHF Symptoms	
Adherence to diet	7		
Health literacy	1		
Hospital admissions	160		

Mortality	105
Value for money	5

Draft
